# The Deletion of the MGF360-10L/505-7R Genes of African Swine Fever Virus Results in High Attenuation but No Protection Against Homologous Challenge in Pigs

**DOI:** 10.3390/v17020283

**Published:** 2025-02-18

**Authors:** Linlin Zheng, Zilong Yan, Xiaolan Qi, Jingjing Ren, Zhao Ma, Huanan Liu, Zhao Zhang, Dan Li, Jingjing Pei, Shuqi Xiao, Tao Feng, Xinglong Wang, Haixue Zheng

**Affiliations:** 1 State Key Laboratory for Animal Disease Control and Prevention, Lanzhou Veterinary Research Institute, College of Veterinary Medicine, Lanzhou University, Chinese Academy of Agricultural Sciences, Lanzhou 730046, China; linlinzheng0605@163.com (L.Z.); qixiaolan@caas.cn (X.Q.); renjingjing@caas.cn (J.R.); liuhuanan2008@163.com (H.L.); zzhao0829@163.com (Z.Z.); lidan@caas.cn (D.L.); jpei@caas.cn (J.P.); shqxiaojd@126.com (S.X.); 2 College of Veterinary Medicine, Northwest A&F University, Yangling 712199, China; 3 Gansu Province Research Center for Basic Disciplines of Pathogen Biology, Lanzhou 730046, China; 4 College of Veterinary Medicine, South China Agricultural University, Guangzhou 510642, China; zilong00scau@163.com; 5 State Key Laboratory of Agrobiotechnology, College of Biological Sciences, China Agricultural University, Beijing 100193, China; mazhaomz@foxmail.com

**Keywords:** ASFV, MGF360-10L, MGF505-7R, attenuated strain, immunoprotection

## Abstract

African swine fever virus (ASFV) is the causative agent of African swine fever (ASF), a severe hemorrhagic disease with a mortality rate reaching 100%. Despite extensive research on ASFV mechanisms, no safe and effective vaccines or antiviral treatments have been developed. Live attenuated vaccines generated via gene deletion are considered to be highly promising. We developed a novel recombinant ASFV strain by deleting *MGF360-10L* and *MGF505-7R*, significantly reducing virulence in pigs. In the inoculation experiment, pigs were infected with 10^4^ 50% hemadsorption doses (HAD_50_) of the mutant strain. All the animals survived the observation period without showing ASF-related clinical signs. Importantly, no significant viral infections were detected in the cohabitating pigs. In the virus challenge experiment, all pigs succumbed after being challenged with the parent strain. RNA-seq analysis showed that the recombinant virus induced slightly higher expression of natural immune factors than the parent ASFV; however, this level was insufficient to provide immune protection. In conclusion, our study demonstrates that deleting *MGF360-10L* and *MGF505-7R* from ASFV CN/GS/2018 significantly reduces virulence but fails to provide protection against the parent strain.

## 1. Introduction

African swine fever (ASF) is a highly contagious and fatal disease caused by African swine fever virus (ASFV) and characterized by fever and hemorrhaging in all organs of pigs. The mortality rate in pigs can reach 100% [1,2] and clinical manifestations resemble those of classical swine fever, including lethargy, loss of appetite, high fever, cyanosis, abortion, edema, hemorrhaging, and ataxia. ASFV also causes immune system disorders in infected animals [3]. ASFV was first reported in Kenya in 1921 and later spread widely among domestic pigs and wild boars across many countries south of the Sahara [4]. Since 2007, ASFV has spread widely in eastern Europe following its introduction into Georgia [5]. In 2018, Rongliang Hu reported the first case of ASF in China [6], and, since then, the epidemic has rapidly spread across the country, threatening the pig farming industry and causing significant economic losses. ASF poses a significant threat to the pig farming industry both in China and worldwide. Currently, no safe and effective vaccine exists, and the epidemic can only be controlled through culling, which not only causes significant economic losses but also causes a failure to meet the needs of large-scale pig farming worldwide [7,8]. Therefore, vaccines, the most effective and economical means of preventing viral infections, are crucial for controlling ASF in pig farming systems.

ASFV is the only known DNA arbovirus and the sole member of the *Asfarviridae* family [9,10,11]. The average diameter of ASFV is 260–300 nm, with a genome size ranging from 170 to 194 kb [11,12]. Structural analysis of ASFV reveals that the viral particle consists of five layers: the outer membrane, the viral capsid, the inner membrane, the nucleocapsid, and the DNA genome [13,14,15,16]. ASFV has a complex structure and a large genome, though most of its functions remain unidentified. The mechanisms underlying ASFV infection and pathogenesis are poorly understood, and the theoretical framework for vaccine development remains limited. Therefore, a promising strategy is to identify virulence genes and develop attenuated ASFV strains. Despite the existence of several attenuated vaccine patents, their safety and efficacy still require further validation.

The development of ASFV vaccines currently involves three main approaches: inactivated vaccines, subunit and live vector vaccines, and recombinant virus vaccines. Among these, inactivated vaccines fail to provide effective immune protection in pigs [17,18], while subunit and live vector vaccines are insufficient to induce robust immunity [19,20,21,22,23,24]. The most promising approach is the gene knockout vaccine, which involves deleting immunosuppressive, tolerance, and ADE-related toxicity genes. This results in vaccine candidates that are both immunogenic and less pathogenic, providing immune protection to pigs.

The deletion of virulence genes is a strategy for constructing attenuated ASFV, and selecting appropriate genes for co-deletion is also critical. Not all combinations of virulence genes lead to effective attenuation and protection. The ASFV genome contains five members of the multigene family (MGF) that have evolved through gene duplication [25]. The co-deletion of MGF505/530 family genes and MGF360 family genes has been shown to provide some degree of attenuation and protection [26]. The deletion of *CD2v*, combined with all MGF360/505 genes, leads to attenuated strains that provide partial immune protection [27], although the virus can still be detected in the serum of immunized pigs. Similarly, the deletion of *MGF360-10L*, *-11L*, *-12L*, *-13L*, and *-14L* along with *MGF505-1R*, *-2R*, and *-3R* from the virulent type II Georgia 2007/1 isolate reduces viral replication in pig macrophages but only provides 25% protection [28]. Furthermore, the joint deletion of *MGF360-12L*, *MGF505-1R*, and *K145R* significantly reduces viral infection in pigs, but only 30% of immunized pigs were protected [28]. In summary, these deletion strains still present safety and efficacy concerns.

In this study, we developed a safer attenuated ASFV strain by deleting *MGF360-10L* and *MGF505-7R* genes. In a high-dose inoculation experiment (104 HAD50), pigs (*n* = 9) showed no signs of disease, indicating that the virus strain did not cause illness or death. Importantly, no significant viremia or ASFV p30-specific antibody response was detected in cohabitating pigs. However, the attenuated virus strain did not confer protection against the high-pathogenic ASFV CN/GS/2018 strain.

## 2. Materials and Methods

### 2.1. Ethics Statements and Safety Protocols

This study was conducted following the guidelines in the Guide for the Care and Use of Laboratory Animals issued by the Ministry of Science and Technology, People’s Republic of China. All animal experiments with ASFV CN/GS/2018 and ASFV-ΔMGF360-10L/Δ505-7R (ASFV-Δ10L/Δ7R) infections were carried out in biosafety-level-3 (P3) facilities at the Lanzhou Veterinary Research Institute (LVRI), Chinese Academy of Agricultural Sciences (CAAS), and approved by the Ministry of Agriculture and Rural Affairs and China National Accreditation Service for Conformity Assessment(permit no. LVRIAEC-2022-024) and date of approval is 28 October 2022.

### 2.2. Cells

The porcine bone marrow-derived macrophages (BMDMs) cell culture was prepared in-house. The protocol was slightly modified from a previously described method [29]. In brief, porcine bone marrow cells were collected from 6~8-week-old piglets that were a commercial hybrid of Duroc × Large White × Landrace with an average weight of 30–40 kg. By using grinding, filtration, washing, and Ficoll density centrifugation, cells were cultured in RPMI 1640 medium (20% FBS, 2% PSN; Thermo Fisher, Waltham, MA, USA) supplemented with 10 ng/mL recombinant porcine granulocyte-macrophage colony-stimulating factor (GM-CSF) (Kingfisher; Beijing, China) to differentiate them into macrophages. After 7 days of differentiation, mature BMDMs were frozen in 90% fetal bovine serum (FBS; CELL-BOX, Changsha, China) and 10% dimethyl sulfoxide (DMSO; Solarbio, Beijing, China) and stored in liquid nitrogen until further use. All cells were incubated at 37 °C with 5% CO_2_.

### 2.3. Viruses and Infection

ASFV strain CN/GS/2018 and the recombinant ASFV-Δ10L/Δ7R were characterized and stored in our laboratory. The virus was propagated in BMDM cells and the supernatants from the infected cells were clarified and stored at −80 °C.

*For viral infection:* Cells were cultured to approximately 80% confluence and infected with ASFV at a specific MOI, as per the experimental design. A mock infection was conducted using phosphate-buffered saline (PBS; Solarbio, Beijing, China). After 2 h of adsorption, the inoculum was removed by aspiration and the cells were washed twice with PBS before incubation in complete medium at 37 °C for various time points until harvesting.

*For the HAD_50_ assay:* The HAD_50_ assay was used to quantify the median tissue culture infectivity of ASFV-WT- and ASFV-Δ10L/Δ7R-infected samples, as described previously, with minor modifications [30]. Briefly, BMDM cells were seeded in 96-well plates for 2 h and 10-fold serially diluted samples were added to each well in triplicate. Next, the cells were incubated with 2% porcine red blood cells diluted in PBS and the hemadsorption (HAD) phenomenon was observed for 5 to 7 days. Finally, HAD_50_ was calculated using the Reed and Muench method [31].

### 2.4. Antibodies

Primary antibody against GAPDH (Beyotime, Shanghai, China) was purchased from Beyotime. The antibodies against p72, p54, and p30 were derived from our laboratory.

### 2.5. Generation of MGF360-10L/MGF505-7R-Deficient Virus

The gene-deleted ASFV-Δ10L/Δ7R mutant was generated by two homologous recombinations of the genome of the parent ASFV CN/GS/2018 isolate with the recombinant transfer vector through transfection and infection procedures. Firstly, the *MGF360-10L* gene in the 1038 bp region was replaced by the reporter gene cassette p72eGFP containing 917 bp, and the obtained recombinant ASFV-ΔMGF360-10L mutant underwent a second recombinant mutation after a series of limited dilution purifications on a monolayer BMDM culture. Similarly, a reporter gene cassette encompassing 908 bp p72mCherry was used to replace the *MGF505-7R* gene in the 1584 bp region and the resulting virus was purified by the continuous selection of fluorescent plaques bound to a limited dilution on a single layer of BMDM. Finally, a double-gene deletion recombinant ASFV-Δ10L/Δ7R mutant expressing eGFP/mCherry signal was obtained. In order to ensure the purity and correct deletion of the recombinant genome, DNA was extracted from the virus stock for PCR analysis. The detailed sequence of primer pairs is shown in Table 1. After the mutated virus was purified, we amplified the virus three times to obtain virus stock (~80 mL), which was aliquoted and frozen in −80 °C freezer.

### 2.6. Whole-Genome Sequencing

The CTAB/SDS method was employed to extract genomic DNA from ASFV strains. DNA concentration and purity were assessed using 1% agarose gel. Genomic DNA was digested with restriction enzymes to create fragments. Barcode ligation was performed on the fragments to prepare for sequencing. The DNA fragments were used to create a sequencing library via PCR amplification. Paired-end sequencing (PE150 mode) was performed on the Illumina HiSeq platform and data analysis was performed according to previously established protocols (34).

### 2.7. qPCR

After infection, we purified total DNA using the E.Z.N.A. Viral DNA Kit (OMEGA, Norcross, GA, USA) following the manufacturer’s instructions. We then performed the TaqMan quantitative PCR assay with the Pro Taq HS Premix Probe qPCR Kit (ACCURATE BIOLOGY AG, Changsha, China) to quantify the virus genome copies. The primers and probes targeting the p72 gene are listed in Table 1. Each 25 μL reaction consisted of 12.5 μL of 2× mix buffer, 3 μL of the standard plasmid/DNA template, 6.5 μL of H_2_O, and 3 μL of the p72 TaqMan probe. The qPCR was conducted using Applied Biosystems QuantStudio 5 as follows: enzyme activation at 95 °C for 2 min (1 cycle) followed by denaturation at 95 °C for 7 s and annealing at 60 °C for 12 s (3 cycles). Finally, denaturation occurred at 95 °C for 6 s and annealing at 58 °C for 11 s (40 cycles). We prepared the standard curve by diluting the standard plasmid ASFV p72 with a known copy number into different gradients. The diluted standard plasmid was incorporated into each of the 24 reactions, with 3 replicates for each sample. The final ASFV genome copy number was analyzed using QuantStudio™ Design & Analysis Software v.1.3.1(©2016 Thermo Fisher Scientific, Waltham, MA, USA).

### 2.8. Real-Time PCR (RT-PCR)

Total RNA was extracted from ASFV-infected cells using TRIzol (Vazyme, Nanjing, China) and then the RNA was reverse-transcribed into cDNA utilizing a reverse transcription kit (Takara, Osaka, Osaka Prefecture, Japan). Finally, according to the instructions of TB Green dye standard quantitative kit (Takara, Osaka, Osaka Prefecture, Japan), qRT-PCR was used to detect mRNA levels of genes. The expression levels of genes were standardized by GAPDH and the relative mRNA expression levels of target genes were evaluated and calculated by the 2^−ΔΔCT^ method. All quantitative primers used in the qRT-PCR assay are shown in Table 1.

### 2.9. Immunoblotting Assay

BMDMs were infected with parental ASFV CN/GS/2018 and ASFV-Δ10L/Δ7R at an MOI of 0.1 for 0, 24, and 48 h. After treatment, cells were washed with cold PBS and incubated on ice with NP-40 lysis buffer (Beyotime, Shanghai, China). Cell lysates were then clarified by centrifugation at 14,000 rpm for 20 min at 4 °C. Proteins were separated by SDS-PAGE and transferred onto polyvinylidene fluoride (PVDF) membranes (Cytiva, The Danaher Corporation, Washington, DC, USA). After treatment with the blocking buffer, the membrane was incubated with primary antibodies against targeted proteins overnight at 4 °C and then with the corresponding HRP-conjugated secondary antibodies at room temperature for 1 h. Protein expression was detected using a chemiluminescent HRP substrate (Merck-Millipore, Billerica, MA, USA). The blot images were obtained from an Amersham Imager 800 (GE Healthcare, Bangalore, India).

### 2.10. Transcriptome Sequencing (RNA-seq) and Analysis

BMDM cells were infected with ASFV CN/GS/2018 or ASFV-Δ10L/Δ7R at 0 h, 12 h, and 24 h and then collected for RNA extraction using TRIzol reagent (Vazyme, Nanjing, China). The RNA integrity and quality were assessed using an Agilent 2100 Bioanalyzer (Agilent Technologies, Santa Clara, CA, USA) with a value of at least 7. Qualified total RNA was used as the starting sample, and the poly(A)-rich RNA in eukaryotic total RNA was enriched using the TIANSeq mRNA capture kit (TIANGEN Biotech, New York, NY, USA). The transcriptome sequencing library was constructed using the TIANSeq rapid RNA library construction kit (TIANGEN Biotech, Illumina platform). After library construction, the library was initially quantified using Qubit2.0, diluted to 1 ng/uL, and then the insert size was detected using an Agilent 2100 Bioanalyzer. After the insert size was confirmed, the effective concentration of the library was accurately quantified using Q-PCR (library effective concentration > 2 nM) to ensure library quality. The different libraries were pooled according to their effective concentrations and the target sequencing data volume requirements and then sequenced using the Illumina platform for PE150 sequencing to obtain 150 bp paired-end sequencing reads. Finally, the differentially expressed genes (DEGs) between ASFV CN/GS/2018- and ASFV-Δ10L/Δ7R-infected BMDM cells with a fold change of ≥2 and a *p*-value < 0.05 were identified using DESeq2 (version 1.0.19) [32]. Finally, the DEGs were enriched for KEGG and GO terms using the Biomart database.

### 2.11. Animal Experiments

The virulence and pathogenic mechanisms of ASFV-Δ10L/Δ7R compared to ASFV CN/GS/2018 were evaluated using mature commercial breeds of pigs. Different groups of pigs were inoculated with 10^4^ HAD_50_ of ASFV-CN/GS/2018 or ASFV-Δ10L/Δ7R. In the protection experiment, the experimental animal group (*n* = 3) was infected with 10^2^ HAD_50_ of the parental virus ASFV-CN/GS/2018 on day 20 to observe the immune protection provided by the deletion virus. In both experimental stages, the duration was 20 days, and clinical symptoms were monitored every day for both control and experimental groups, including body temperature and mortality rate. Meanwhile, blood was collected from the jugular vein, and serum was separated to detect the expression of viral DNA and anti-p30 antibodies. At the end of the experiment, the hearts, livers, spleens, lungs, kidneys, and lymph nodes of dead pigs or surviving pigs were collected. According to the requirements of the World Organisation for Animal Health (WOAH), surviving pigs were euthanized and their blood and tissues were safely disposed of to prevent the release of ASFV [33].

### 2.12. Blocking ELISA

According to the instructions of the Block ELISA kit (Lanzhou Shouyan Biotechnology Co., Ltd.; Lanzhou, China), ASFV p30 specific antibody was detected in serum. When the colorimetric reaction was terminated, the results of the analysis of the optical density (OD_450_) value at 450 nm were read on an enzyme-labeled instrument, and the closure rate was calculated based on the standard curve independently established for each experimental datum. The cut-off value was 40.

### 2.13. Statistical Analysis

Statistical analysis was performed using GraphPad Prism 10, with data presented as mean values ± standard errors of the mean (SEMs). A *p*-value < 0.05 was considered statistically significant.

## 3. Results

### 3.1. ASFV-Δ10L/Δ7R Exhibited Lower Replication Capacity than ASFV CN/GS

**Figure 1:**
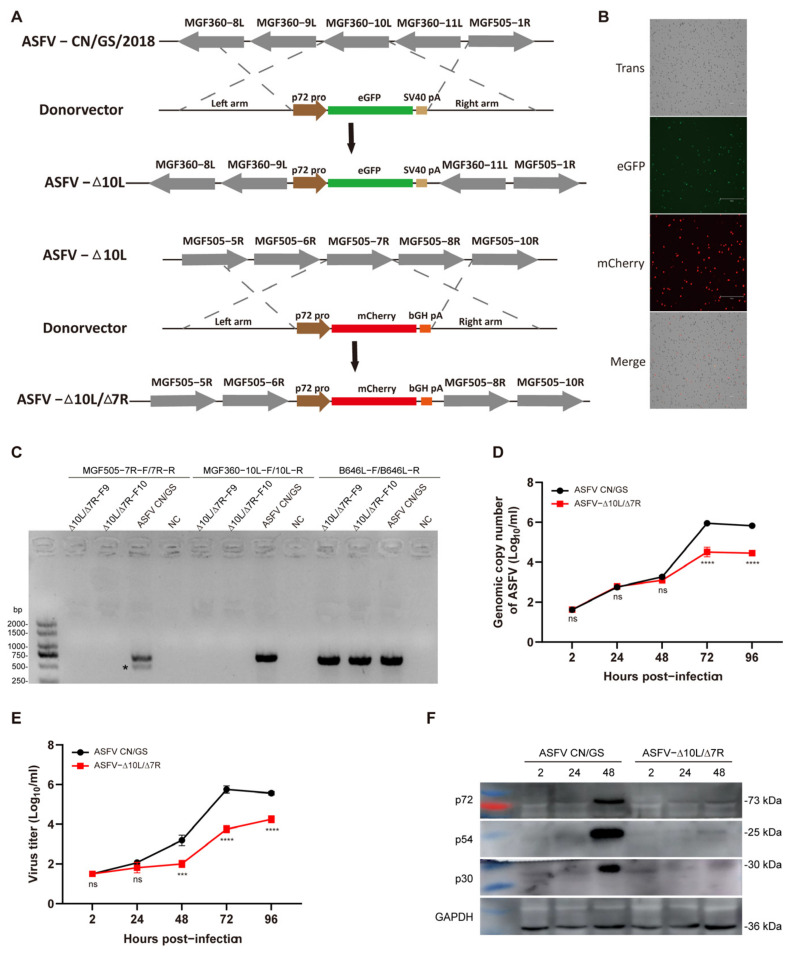
Construction and growth dynamics of the ASFV-Δ10L/Δ7R mutant. (**A**) Schematic diagram of the deletion of the *MGF360-10L* and *MGF505-7R* gene regions in ASFV CN/GS/2018. The *MGF360-10L* sequence was replaced with a p72-eGFP reporter gene cassette. The *MGF505-7R* sequence was replaced with a p72-mCherry reporter gene cassette in the ASFV-ΔMGF360-10L deletion strain. (**B**) Fluorescent expression in ASFV-Δ10L/Δ7R-infected BMDMs. Scale bar: 300 μm. (**C**) PCR verification of the deletion virus genome using specific primers for *MGF505-7R*, *MGF360-10L*, and the positive control *B646L* (p72). ∗, nonspecific bands. (**D**) Viral DNA replication of ASFV CN/GS and ASFV-Δ10L/Δ7R. BMDMs were infected with ASFV CN/GS or ASFV-Δ10L/Δ7R (0.01 MOI) and viral genome copies were quantified at different time points. (**E**) Growth curves of ASFV CN/GS/2018 and ASFV-Δ10L/Δ7R. BMDMs were infected with ASFV CN/GS or ASFV-Δ10L/Δ7R (0.01 MOI) and viral titers were determined by red blood cell hemagglutination assays at various time points. (**F**) Expression of p72, p54, and p30 in ASFV CN/GS or ASFV-Δ10L/Δ7R at different time points. BMDMs were infected with ASFV CN/GS or ASFV-Δ10L/Δ7R (0.1 MOI), respectively. Samples were collected at 2, 24, and 48 h post-infection and Western blotting was performed. Data represent at least three independent experiments (**D**–**F**). [(**D**,**E**)] are presented as mean ± SEM of three biological replicates and were analyzed by one-way ANOVA. (**F**) Data are representative of three independent experiments. ns for *p* > 0.05, *** *p* < 0.001, **** *p* < 0.0001.

Numerous studies have highlighted the critical role of the MGF360 and MGF505 gene families in ASFV pathogenicity. Deletions in MGF360 and/or MGF505, along with other genes, have been shown to reduce the virulence of parent strains and provide immune protection in recombinant viral vaccines [26,27,28,34]. To assess the effects of deleting *MGF360-10L* and *MGF505-7R* genes on ASFV CN/GS/2018, a genotype II strain endemic in China, we constructed ASFV-Δ10L/Δ7R using homologous recombination (Figure 1A). The *MGF360-10L* sequence was replaced with a p72eGFP reporter gene cassette and the virus was purified using porcine BMDM. The MGF505-7R sequence was then replaced with a p72mCherry reporter gene cassette, successfully generating the ASFV-Δ10L/Δ7R mutant, which was verified by fluorescence (Figure 1B). PCR analysis was performed using specific primers targeting the deletion sequence and B646L primers (internal control). No band was detected in the ASFV-Δ10L/Δ7R stock, confirming successful purification from the wild-type strain (Figure 1C). To evaluate potential off-target effects of the homologous recombination strategy used to generate the ASFV-GS-Δ7R/ΔI267L strain, the whole genomes of both the ASFV-GS-Δ7R/ΔI267L mutant and its parental strain were sequenced. Apart from the mutations at the targeted sites, the analysis revealed only four single nucleotide variations (SNVs) between the two genomes. These SNVs were located in the genes *M1249L*, *D205R*, and *E199L* (Table 2). These variations appeared to result from viral replication and were unlikely to cause significant changes in viral virulence.

To assess the growth kinetics of ASFV-Δ10L/Δ7R in vitro, BMDM cells were infected with 0.01 MOI of ASFV CN/GS or ASFV-Δ10L/Δ7R. Viral genome copy numbers were measured at various time points post-infection. No significant differences were observed between ASFV and ASFV-Δ10L/Δ7R within the first 48 h of infection. However, after 48 h, the viral genome copy number of ASFV-Δ10L/Δ7R was significantly lower (Figure 1D) and its virus titer was also reduced compared to ASFV CN/GS (Figure 1E). Comparable results were obtained when measuring viral protein levels in infected cells (Figure 1F), further confirming that ASFV-Δ10L/Δ7R had a lower replication capacity than the parent strain.

### 3.2. ASFV-Δ10L/Δ7R Exhibited Significant Attenuation in Pigs

**Figure 2:**
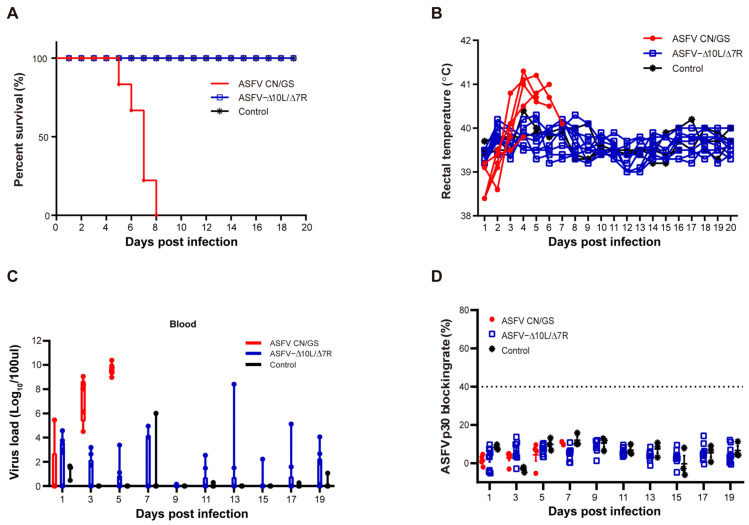
Evaluation of the virulence of ASFV-Δ10L/Δ7R at the animal level. (**A**) Pigs were injected intramuscularly with ASFV CN/GS or ASFV-Δ10L/Δ7R (10⁴ HAD_50_), followed by analyses of survival rates (**A**), thermodynamics (**B**), viral load in the blood (**C**), and p30-specific antibody levels (**D**). [(**C**,**D**)] Data are presented as mean ± SEM of three biological replicates.

To evaluate the virulence of ASFV-Δ10L/Δ7R in vivo, two groups of pigs were inoculated with 10^4^ HAD_50_ of ASFV-Δ10L/Δ7R (*n* = 9) or ASFV CN/GS (*n* = 5), while a control group (*n* = 3) was housed with the ASFV-Δ10L/Δ7R-infected group. Clinical indicators, including temperature, were monitored and recorded for 20 days post-infection (dpi). As shown in Figure 2A, pigs in the ASFV CN/GS-infected group succumbed sequentially before 8 dpi, with body temperatures exceeding 40 °C by 3 dpi (Figure 2B). In contrast, no deaths or significant increases in body temperature were observed in the ASFV-Δ10L/Δ7R-infected group. Furthermore, the typical clinical signs of ASF were absent in the cohabitant pigs (Figure 2B). These results indicate that the deletion of the MGF360-10L and MGF505-7R genes completely attenuated the virulence of ASFV CN/GS. Viral load was assessed at different time points (Figure 2C), showing a high viral load in the blood of ASFV CN/GS-infected pigs, reaching approximately 10^10^ HAD_50_/100 μL prior to death. In contrast, the viral load in ASFV-Δ10L/Δ7R-infected pigs was generally below 10^3^ HAD_50_/100 μL, with even lower levels observed in the control group. While the immune mechanisms induced by ASFV CN/GS remain unclear, it is also uncertain whether infection with attenuated strains induces immune responses that confer protection against virulent strains. However, as is well established, humoral immunity plays a critical role in virus clearance [35,36,37]. To assess the antibody response, we measured anti-p30 circulating antibody levels using a blocking ELISA. The results revealed a gradual increase in the p30 blocking rate following ASFV CN/GS infection, reaching a peak before death; however, the maximum value was far below the cut-off threshold. No significant difference in antibody levels was observed between ASFV-Δ10L/Δ7R-infected and control groups (Figure 2D), suggesting that ASFV-Δ10L/Δ7R did not induce a significant immune response in animals.

### 3.3. ASFV-Δ10L/Δ7R Did Not Induce Immune Protection Against Challenge with the Parental Strain

**Figure 3:**
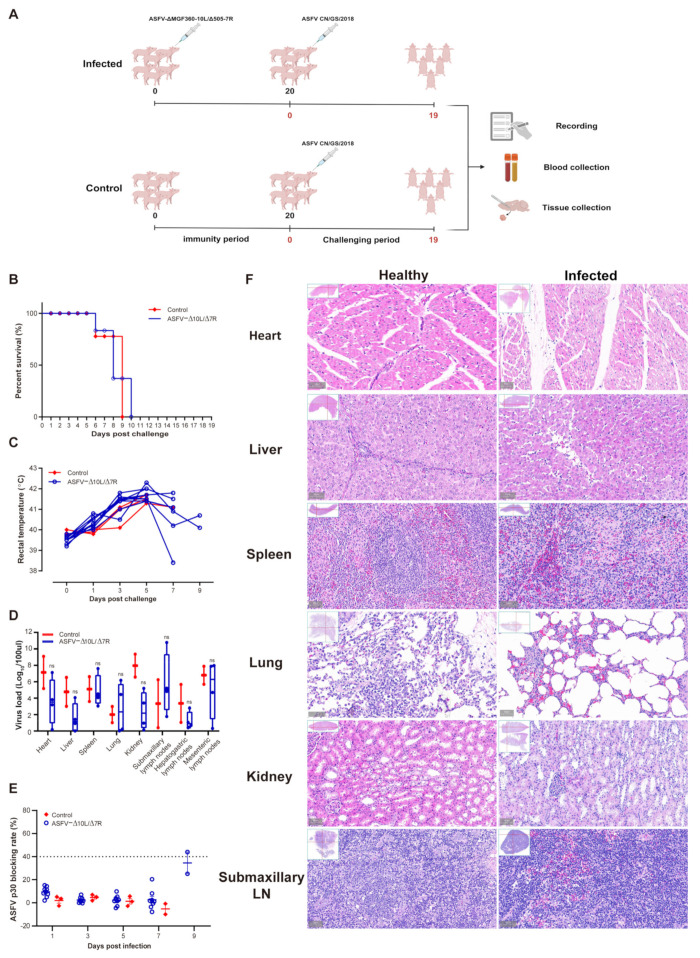
The immune protection response of pigs induced by ASFV CN/GS challenge. (**A**) Immunization and challenge model: 10⁴ HAD_50_ of ASFV-Δ10L/Δ7R was injected intramuscularly into pigs, followed by a challenge with 10^2^ HAD_50_ of ASFV CN/GS 20 days later. Animal parameters were evaluated at different time points after challenge, including survival rate (**B**), thermodynamics (**C**), viral load in various tissues (**D**), and p30-specific antibody levels (**E**). (**F**) Comparison of characteristic histopathological changes between the healthy group and ASFV-Δ10L/Δ7R-infected animals after challenge with ASFV CN/GS. [(**D**,**E**)] Data are presented as mean ± SEM of three biological replicates and (**D**) was analyzed by two-way ANOVA. ns for *p* > 0.05.

To evaluate the immune protection conferred by ASFV-Δ10L/Δ7R, we challenged animals with the parental virus after inoculation with the attenuated strain. As shown in Figure 3A, the experimental group (*n* = 9) was initially injected intramuscularly with 10^4^ HAD_50_ of ASFV-Δ10L/Δ7R, followed by a challenge with 10^2^ HAD_50_ of ASFV CN/GS at 20 dpi. Clinical symptoms and body temperature changes were monitored daily. In the ASFV-Δ10L/Δ7R group, deaths began at 6 days post-challenge (dpc) with ASFV CN/GS and were completed by 10 dpc, accompanied by an increase in body temperature, similar to the control group (*n* = 3), which was infected solely with ASFV (Figure 3B,C). All animals in the ASFV-Δ10L/Δ7R group were susceptible to challenge by the highly pathogenic parental strain. Viral gene copy numbers in various tissues, including the heart, liver, spleen, lungs, kidneys, submaxillary lymph nodes, hepato-gastric lymph nodes, and mesenteric lymph nodes, were not significantly different between ASFV-Δ10L/Δ7R-infected animals and those infected with the parental strain (Figure 3D). Similarly, anti-p30 circulating antibody levels did not show a significant increase (Figure 3E), indicating that ASFV-Δ10L/Δ7R failed to induce adequate immune protection in the host.

Histopathological examination (Figure 3F) revealed extensive pathological damage in the challenged group. Cardiac muscle fibers appeared deformed, liver cell arrangement was disrupted with loosely arranged cells and significant inflammatory cell infiltration, the red and white pulp of the spleen were disordered and loosely arranged, lung tissue exhibited edema and disorganized alveolar structures, renal tubules showed vacuole-like degeneration with increased interstitial space and loose arrangement, and submaxillary lymphoid tissue exhibited blurred gland boundaries, a marked reduction in blood vessels, and widespread red blood cell exudation across all tissues. These findings suggest that ASFV-Δ10L/Δ7R infection does not provide protection against a subsequent challenge by ASFV CN/GS, as evidenced by the lack of protective effects on tissue integrity.

### 3.4. Transcriptome Analysis of BMDM Cells Infected with ASFV CN/GS and ASFV-Δ10L/Δ7R

**Figure 4:**
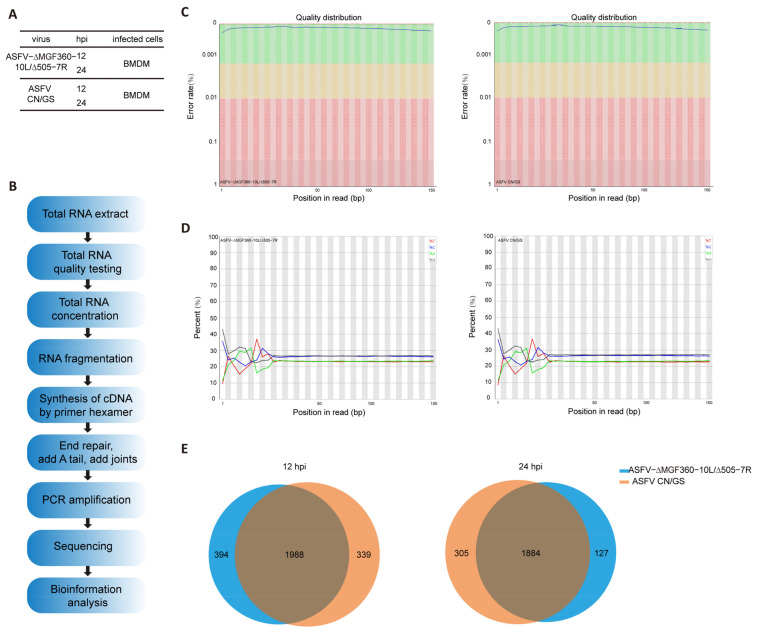
Experimental design and quality assessment of RNA-seq for ASFV-Δ10L/Δ7R. [(**A**,**B**)] Experimental design and RNA-seq sequencing process. (**A**) BMDM cells were infected with 0.1 MOI of ASFV CN/GS or ASFV-Δ10L/Δ7R, and samples were collected at 12 h and 24 h for analysis. (**B**) Samples from (**A**) were prepared and processed for bioinformatics sequencing. [(**C**,**D**)] Quality distribution (**C**) and base composition (**D**) of the original sequencing data for ASFV CN/GS and ASFV-Δ10L/Δ7R. (**E**) Venn diagram showing differentially expressed genes (DEGs) in ASFV CN/GS and ASFV-Δ10L/Δ7R at different infection times.

**Figure 5:**
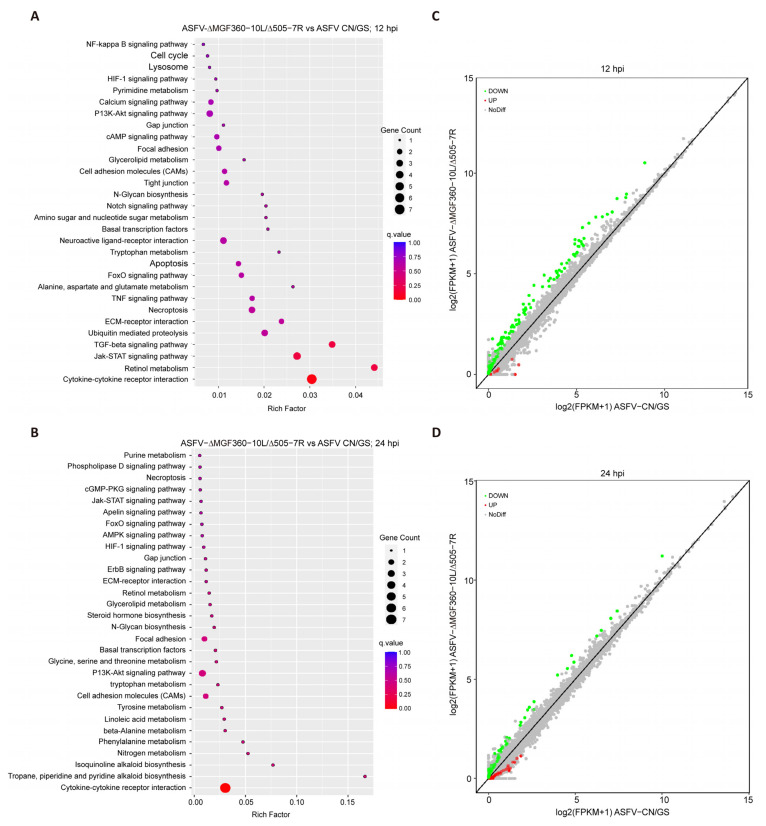
Analysis of KEGG pathway and scatter plot for DEGs. [(**A**,**B**)] KEGG pathway analysis of DEGs (*p* < 0.05 and |log2FC| > 1) in cells infected with ASFV CN/GS or ASFV-Δ10L/Δ7R at 12 h and 24 h. KEGG enrichment was measured by RichFactor, *p*-value, and the number of genes associated with each pathway. The color and size of the dots represent fold changes and the number of DEGs enriched in each pathway, respectively. [(**C**,**D**)] Scatter plot analysis of DEGs (*p* < 0.05 and |log2FC| > 1) in cells infected with ASFV CN/GS or ASFV-Δ10L/Δ7R at 12 h and 24 h. In the differential expression scatter plot, each dot represents a gene: green dots indicate downregulated DEGs, red dots indicate upregulated DEGs, grey dots represent genes with no significant difference, and the diagonal line represents genes with equal expression in both groups.

To further investigate the host cell response to ASFV CN/GS and ASFV-Δ10L/Δ7R infection, BMDM cell cultures were collected at various time points post-infection (Figure 4A), and RNA sequencing was performed as outlined in the workflow (Figure 4B). The sequencing data quality and base distributions for ASFV-Δ10L/Δ7R (left) and ASFV CN/GS (right) met experimental standards (Figure 4C,D). Figure 4E shows the number of differentially expressed genes (DEGs) for ASFV-Δ10L/Δ7R (2382 at 12 hpi and 2011 at 24 hpi) and ASFV CN/GS (2327 at 12 hpi and 2189 at 24 hpi) with statistical significance (*p* < 0.05, |log2FC| > 1). Notably, the expression of innate immune factors was higher in ASFV-Δ10L/Δ7R-infected cells than in those infected with the parental strain, suggesting that ASFV-Δ10L/Δ7R may induce a stronger host immune response. To investigate changes in biological signaling pathways, we performed KEGG pathway analysis of the DEGs from cells infected with ASFV-Δ10L/Δ7R and ASFV CN/GS at different time points. The analysis revealed that most DEGs were associated with immune-related pathways (Figure 5A,B), including NF-κB and JAK/STAT signaling. Scatter plot analysis further demonstrated that 138 and 67 DEGs were significantly downregulated at 12 and 24 hpi, respectively, while 9 and 40 DEGs were significantly upregulated (Figure 5C,D).

### 3.5. ASFV-Δ10L/Δ7R-Induced Upregulation of Immune Response-Related Factors Compared to ASFV CN/GS

**Figure 6:**
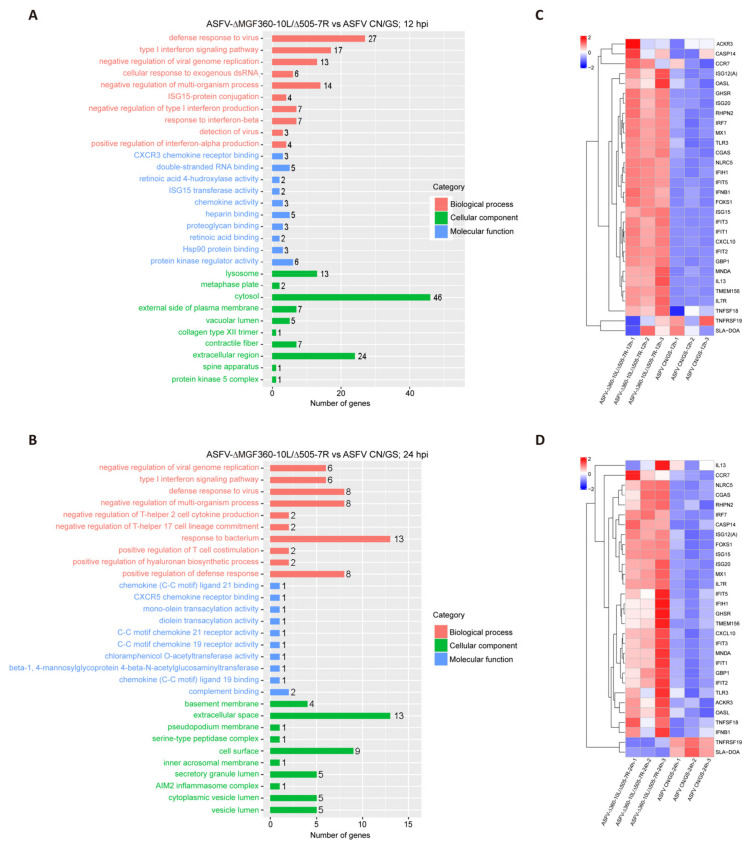
Comparison of RNA-seq results between ASFV CN/GS and ASFV-Δ10L/Δ7R during infection. [(**A**,**B**)] Gene Ontology (GO) functional enrichment analysis of DEGs in cells infected with ASFV CN/GS or ASFV-Δ10L/Δ7R at 12 h and 24 h. [(**C**,**D**)] Heatmap of DEGs in cells infected with ASFV CN/GS or ASFV-Δ10L/Δ7R at 12 h and 24 h. A total of 30 DEGs related to innate immunity were selected and are displayed in the heatmap. The colors represent the fold changes (FCs) at the indicated time points.

**Figure 7:**
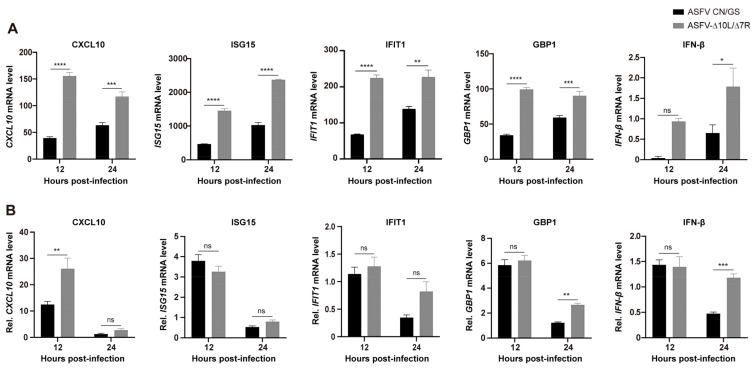
Expression levels of innate immune-related DEGs. (**A**) RNA-Seq analysis of the expression levels of *CXCL10*, *ISG15*, *IFIT1*, *GBP1*, and *IFN-β* genes in BMDMs infected with ASFV CN/GS or ASFV-Δ10L/Δ7R at 12 h and 24 h. (**B**) RT-qPCR analysis of the relative mRNA expression levels of *CXCL10*, *ISG15*, *IFIT1*, *GBP1*, and *IFN-β* genes in ASFV CN/GS- and ASFV-Δ10L/Δ7R-infected BMDMs (0.1 MOI) at 12 h and 24 h. [(**A**,**B**)] Data represent at least three independent experiments and are presented as mean ± SEM of three biological replicates analyzed by two-way ANOVA. ns for *p* > 0.05, * *p* < 0.05, ** *p* < 0.01, *** *p* < 0.001, **** *p* < 0.0001.

To further analyze the function of DEGs in host responses, we conducted a Gene Ontology (GO) functional analysis and selected the top 10 most enriched terms. The results revealed that the immune response was the most significantly enriched biological process at 12 hpi (Figure 6A). Although the immune response decreased at 24 hpi (Figure 6B), it remained predominantly focused on the innate immune, the first line of defense. We selected thirty immune-related DEGs and visualized their expression profiles using a heatmap (Figure 6C,D). To verify these findings, qRT-PCR was performed to assess the expression of five key DEGs (Figure 7A,B). RNA sequencing analysis (Figure 7A) showed that the expression levels of *CXCL10*, *ISG15*, *IFIT1*, *GBP1*, and *IFN-β* were generally higher in ASFV-Δ10L/Δ7R-infected cells compared to ASFV CN/GS-infected cells (Figure 7B). As expected, mRNA levels of all five genes were upregulated in BMDM cells infected with ASFV-Δ10L/Δ7R, confirming the RNA sequencing results (Figure 7B).

## 4. Discussion

Vaccine development based on viral genome modification is an active research field and plays a critical role in combating ASFV. In this study, we generated a novel mutant strain by deleting the *MGF360-10L* and *MGF505-7R* genes from the ASFV CN/GS/2018 genome. Our findings demonstrate that this mutant was completely attenuated in pigs but did not induce immune protection against a homologous challenge. Additionally, we observed differential cellular responses of BMDMs to ASFV-Δ10L/Δ7R and its parental strain.

Safety and efficacy are two critical factors in vaccine development, and both must be carefully balanced. Currently, the roles of ASFV virulence genes are not fully understood, and no single-gene deletion has been shown to meet both safety and efficacy requirements. A common approach involves targeting multiple genes. Previous studies have demonstrated that deleting the MGF360 and/or MGF505 family genes significantly attenuates ASFV strains and provides immune protection against the wild-type virus. These genes have been implicated in virulence attenuation and the suppression of IFN-I responses [38,39,40,41,42,43,44]. However, their roles may vary between different ASFV isolates, and the impact of this variability on ASFV pathogenesis remains understudied. Deleting MGF505-7R in the ASFV HLJ/18 strain increases IL-1β and IFN-I production [45], while the MGF505-7R gene from ASFV CN/GS inhibits IFN-II levels through the JAK-STAT signaling pathway [46]. These findings suggest that MGF505-7R plays a key role in ASFV virulence. Although the mechanism of *MGF360-10L* remains unclear, deleting MGF360 family genes in the Benin 97/1 strain elevates IFN-β levels and confers protection against parental strain challenge [26]. In this study, we constructed attenuated strains by deleting *MGF360-10L* and *MGF505-7R* from ASFV CN/GS/2018.

In vitro, ASFV-Δ10L/Δ7R showed significantly reduced replication capacity. The genome copy number decreased significantly at 72 hpi compared to the parental strain, with a notable decrease in viral titers at 48 hpi. This early decline may result from the deletion of these genes, which impairs both viral genome replication and gene translation. Western blot analysis confirmed the reduced expression of viral proteins p72, p54, and p30 at 48 hpi. These results suggest that MGF360-10L and MGF505-7R play key roles in viral genome replication and protein synthesis and potentially other stages of the ASFV life cycle, such as particle assembly or budding.

However, the virus challenge results indicate that ASFV-Δ10L/Δ7R is not a viable vaccine candidate as it failed to induce immune protection against the parental virus. The lack of significant p30-specific antibody production suggests that the mutant failed to elicit a protective ASFV-specific immune response. RNA-seq analysis showed that while innate immune factor expression was upregulated in ASFV-Δ10L/Δ7R-infected cells compared to the parental strain, this response was insufficient to prevent viral replication and protect against challenge. These results indicate that the deletion of *MGF360-10L* and *MGF505-7R* causes excessive attenuation, preventing the mutant from inducing an adequate immune response. An important observation from the inoculation experiment was the absence of significant temperature fluctuations, viremia, or p30-specific antibody responses in cohabiting animals. This suggests that ASFV-Δ10L/Δ7R does not transmit between animals after immunization, providing evidence that it could serve as a safe backbone for the development of more effective live attenuated vaccines (LAVs) against ASFV.

In conclusion, our results show that the ASFV-ΔMGF360-10L/Δ505-7R mutant derived from ASFV CN/GS/2018 is fully attenuated in pigs but fails to elicit a strong immune response, thus providing no protection against the parental strain. These findings provide valuable insights for the development of gene-deleted LAVs for ASFV.

## Figures and Tables

**Table 1:** List of primers and primer sequences.

Primer		Sequence
p72 (Taqman qPCR)	Forward	5’-ATGGAAATTCCCTAGACGAA-3’
	Reverse	5’-CACTGGTTCCCTCCACCGAT-3’
	TaqMan probe	5’-FAM-ACCTCCTGGCCAACCAAGTGCT-BHQ1-3’
p-IFIT1	Forward	5’-TCAGAGGTGAGAAGGCTGGT-3’
	Reverse	5’-GCTTCCTGCAAGTGTCCTTC-3’
p-ISG15	Forward	5’-GACTGCATGATGGCATCGGA-3’
	Reverse	5’-TGCACCATCAACAGGACCAT-3’
p-GBP1	Forward	5’-GAAGGGTGACAACCAGAACGAC-3’
	Reverse	5’-AGGTTCCGACTTTGCCCTGATT-3’
p-INF-β	Forward	5’-CACTGGCTGGAATGAAACCg-3’
	Reverse	5’-AATGGTCATGTCTCCCCTGG-3’
p-CXCL10	Forward	5’-CTGTTCGCTGTACCTGCATC-3’
	Reverse	5’-GCTTCTCTCTGTGTTCGAGG-3’
p-GAPDH	Forward	5’-ACATGGCCTCCAAGGAGTAAGA-3’
	Reverse	5’-GATCGAGTTGGGGCTGTGACT-3’
505-7R	Forward	5’-TTTGGGAAAATCCCGCGGAAAGAA-3’
505-7R	Reverse	5’-TCCTGTAGGGAGAACATTTTCTCT-3’
360-10L	Forward	5’-TAGGGCCTTTGCCTCTTCAAAGG-3’
360-10L	Reverse	5’-GGCTTGGACCTTAATACGGCAC-3’
B646L	Forward	5’-CCGGGGTATTCGCAGTAGTA-3’
B646L	Reverse	5’-ATCCCGAACCCACTTTGAGT-3’

**Table 2:** Summary of mutations between ASFV-Δ10L/Δ7R and its parental strain.

Mutation Type	Start	Ref.	Alt.	Gene	Amino Acid Change
SNV	79065	T	C	M1249L	Asp455Gly
SNV	138786	A	C	D205R	Asn102Thr
SNV	167129	T	G	E199L	Gln104His
SNV	167188	C	G	E199L	Ala85Pro

## Data Availability

The raw data of RNA-seq generated during this study are available on the NCBI BioProject database (https://www.ncbi.nlm.nih.gov/bioproject/) (accessed on 3 December 2024) under BioProject PRJNA1215345. Other data needed to evaluate the conclusions in this paper are present in the paper.
